# The Dose Response Multicentre Investigation on Fluid Assessment (DoReMIFA) in critically ill patients

**DOI:** 10.1186/s13054-016-1355-9

**Published:** 2016-06-23

**Authors:** F. Garzotto, M. Ostermann, D. Martín-Langerwerf, M. Sánchez-Sánchez, J. Teng, R. Robert, A. Marinho, M. E. Herrera-Gutierrez, H. J. Mao, D. Benavente, E. Kipnis, A. Lorenzin, D. Marcelli, C. Tetta, C. Ronco, for the DoReMIFA study group, M Balciunas, B. S. Oliveira, L. Cachafeiro, E. G. Porcile, M. Ranieri, V. Cantaluppi, V. Schweiger, L. Montini, M. Gurjar, C. Liuzzo, N. Brienza, J. Silvestre

**Affiliations:** Department of Nephrology Dialysis and Transplantation, San Bortolo Hospital, 37 Via Rodolfi, 36100 Vicenza, Italy; International Renal Research Institute of Vicenza (IRRIV), San Bortolo Hospital, 37 Via Rodolfi, 36100 Vicenza, Italy; Department of Critical Care, King’s College London, Guy’s and St Thomas’ Hospital, Westminster Bridge Road, London, SE1 7EH UK; Servicio de Medicina Intensiva, Hospital Universitario del Vinalopo, Calle Tonico Sansano Mora, 14, 03283 Elche, Spain; Intensive Care, Hospital Universitario La Paz/Carlos III. IdiPAZ, Paseo Castellana 261, 28046 Madrid, Spain; Department of Nephrology, Shanghai Institute of Kidney and Dialysis, Shanghai Key Laboratory of Kidney and Blood Purification, Zhongshan Hospital, Fudan University, 180 Fenglin Road, 200032 Shanghai, China; Medical Intensive Care, University of Poitiers; CHU Poitiers, 2, rue de la Milétrie, Poitiers, 86021 France; Intensive Care Service, St Antonio Hospital – Porto, Largo Prof. Abel Salazar, 4099-001 Porto, Portugal; Intensive Care Unit, General University Hospital, Avd Carlos Haya s/n, Malaga, 29010 Spain; Department of Nephrology, The First Affiliated Hospital of Nanjing Medical University, 300 Guangzhou Road, 210029 Nanjing, Jiangsu China; Department of Nephrology, Clinica Las Condes, Estoril 450, Las Condes, 7591283 Santiago, Chile; Department of Anesthesiology and Critical Care, University Hospital, EA 7366, Lille, 59000 France; Fresenius Medical Care, Else-Kröner-Straße 1, 61352 Bad Homburg, Germany

**Keywords:** Fluid overload, RRT, AKI, Critical illness, ICU

## Abstract

**Background:**

The previously published “Dose Response Multicentre International Collaborative Initiative (DoReMi)” study concluded that the high mortality of critically ill patients with acute kidney injury (AKI) was unlikely to be related to an inadequate dose of renal replacement therapy (RRT) and other factors were contributing. This follow-up study aimed to investigate the impact of daily fluid balance and fluid accumulation on mortality of critically ill patients without AKI (N-AKI), with AKI (AKI) and with AKI on RRT (AKI-RRT) receiving an adequate dose of RRT.

**Methods:**

We prospectively enrolled all consecutive patients admitted to 21 intensive care units (ICUs) from nine countries and collected baseline characteristics, comorbidities, severity of illness, presence of sepsis, daily physiologic parameters and fluid intake-output, AKI stage, need for RRT and survival status. Daily fluid balance was computed and fluid overload (FO) was defined as percentage of admission body weight (BW). Maximum fluid overload (MFO) was the peak value of FO.

**Results:**

We analysed 1734 patients. A total of 991 (57 %) had N-AKI, 560 (32 %) had AKI but did not have RRT and 183 (11 %) had AKI-RRT. ICU mortality was 22.3 % in AKI patients and 5.6 % in those without AKI (*p* < 0.0001). Progressive fluid accumulation was seen in all three groups. Maximum fluid accumulation occurred on day 2 in N-AKI patients (2.8 % of BW), on day 3 in AKI patients not receiving RRT (4.3 % of BW) and on day 5 in AKI-RRT patients (7.9 % of BW). The main findings were: (1) the odds ratio (OR) for hospital mortality increased by 1.075 (95 % confidence interval 1.055–1.095) with every 1 % increase of MFO. When adjusting for severity of illness and AKI status, the OR changed to 1.044. This phenomenon was a continuum and independent of thresholds as previously reported. (2) Multivariate analysis confirmed that the speed of fluid accumulation was independently associated with ICU mortality. (3) Fluid accumulation increased significantly in the 3-day period prior to the diagnosis of AKI and peaked 3 days later.

**Conclusions:**

In critically ill patients, the severity and speed of fluid accumulation are independent risk factors for ICU mortality. Fluid balance abnormality precedes and follows the diagnosis of AKI.

**Electronic supplementary material:**

The online version of this article (doi:10.1186/s13054-016-1355-9) contains supplementary material, which is available to authorized users.

## Background

Renal replacement therapy (RRT) constitutes a key component of modern critical care, together with mechanical ventilation, fluid resuscitation and vasopressor support. The main reasons for initiation are acute kidney injury (AKI) and fluid overload (FO). There is growing evidence that FO is harmful and associated with a longer hospital stay and increased morbidity and mortality [1–3]. FO may be present at admission or develop during ICU stay due to a combination of oliguria and liberal fluid administration leading to a positive fluid balance [4, 5].

FO accounts for an increased risk of death in patients with AKI [6, 7]. Furthermore, fluid accumulation itself may be independently associated with an increased risk of developing AKI and mortality. In a secondary analysis of the SOAP study, Payen et al. showed that the average daily fluid balance in the first 7 days was significantly more positive in patients with AKI [1]. Bouchard et al. demonstrated that crude mortality was higher in AKI patients when fluid overload was present [8]. Subsequent studies confirmed that both, severity of FO and the number of days spent with FO, were risk factors for poor outcome.

We previously published a prospective cohort observational study, the “Dose Response Multicentre International Collaborative Initiative (DoReMi)” [9], which evaluated the practice of continuous renal replacement therapy (CRRT) in patients in the intensive care unit (ICU). The study confirmed that in spite of a discrepancy (−25 %) between effective (27 ml/kg/h) and prescribed (34 ml/kg/h) dose, the median delivered dose was considered adequate based on two recent large trials (RCTs) [10, 11]. The DoReMi study therefore concluded that the high mortality observed in AKI patients was not related to inadequate treatment dose but to other possible factors instead.

The “Dose Response Multicentre Investigation on Fluid Assessment (DoReMIFA)” study is an evolution of the previous DoReMi study. It aimed to prospectively evaluate the practice of fluid management in the ICU, including patients with AKI (AKI) and without AKI (N-AKI), and patients with AKI treated with RRT (AKI-RRT) in different ICUs (in Europe, the Far East and Latin America). The main objective of this study was to investigate whether fluid balance throughout ICU stay and during RRT affects mortality of ICU patients. Data collection and analysis was facilitated by the use of electronic medical records and web-based case report forms (CRFs).

## Methods

The study protocol was made available for review to ICU physicians from different countries. Once the ICU was enrolled in the research group, data were collected for all admitted patients for 3 consecutive months, in the period between April 2012 and September 2014 using an electronic case report form. Exclusion criteria were (a) age <18 years or >85 years; (b) chronic dialysis; (c) short-term postoperative admission; (d) life expectancy less than 48 h; (e) need for extracorporeal membrane oxygenation (ECMO) within the first 48 h of ICU stay. All types of ICUs were eligible on voluntary basis within the indicated period. Local ethics committees approved the study according to the local regulations.

### Data collection

We designed a specific password-protected website for the study. Centres could only access data related to their patients. An automatic data verification system screened each field for missing or out-of-range values and data inconsistencies, generating a visual alert. The online tool included seven sections: (1) Demographics, anthropometrics, and admission diagnoses; (2) Medical history; (3) Admission data; (4) Daily vital signs and laboratory values; (5) RRT; (6) Sepsis and (7) Outcomes/case closure. Data input was on daily basis. Severity of illness was defined by Simplified Acute Physiology Score II (SAPS II) [12], Sequential Organ Failure Assessment (SOFA) [13] and Acute Physiology and Chronic Health Evaluation II (APACHE II) [14] scores at admission and described in a pie chart (Additional file 1: Figure S1). AKI diagnosis was based on creatinine (Cr) levels using the KDIGO classification [15]. The creatinine value prior to hospitalization (Medical history) was used as baseline. If missing, a reference creatinine was requested utilizing the Modification of Diet in Renal Disease (MDRD) formula assuming a glomerular filtration rate of 75 ml/min/1.73 m^2^. Daily data entry included serum creatinine, total fluid intake and output, urine volume, ventilation modality, diuretic therapy and, if present, RRT. As soon as a patient met the criteria for AKI, a full data entry section was activated for 14 days with automatic daily display of AKI stage. Data for SOFA calculation and for epinephrine/norepinephrine dose were additionally included. A dynamic chart displaying fluid balance and clinical management was generated using the data entered daily (see Additional file 1: Figure S2). The RRT section included data on modality, flows (blood, dialysate, reinfusion, pre/post ratio, and net ultrafiltration rate), anticoagulation, duration (for each 24-h period), reason for initiation, type of vascular access and malfunctions. RRT fields were automatically adjusted according to treatment modality. It was mandatory to report the cause of downtime whenever the effective treatment time was less than that prescribed. Data on circuit life span were recorded for each treatment. The case closure section included data on outcome and renal status at discharge. Data on fluid balance were automatically calculated by the CRF from the time of admission, based on daily data entry.

### Definitions

Fluid overload (FO) was defined as the ratio between cumulative fluid balance and the initial body weight, in percentage. Maximum fluid overload (MFO) referred to the peak value of FO observed during the entire ICU stay. TMFO represented the number of days between ICU admission and day of MFO. Fluid overload slope (FO_SL_) was computed as the ratio MFO/TMFO and represented the velocity of fluid accumulation. The term “AKI-RRT” defined AKI patients who received at least one session of RRT while “AKI” referred to AKI patients without RRT, when not differently specified. N-AKI defined patients who never developed AKI. AKI stages were based on the creatinine criteria of the KDIGO classification.

### Statistical analysis

Continuous variables were described as mean ± standard deviation (SD) or median and interquartile range (IQR). Percentages were calculated for categorical variables. Bivariate comparisons of patients with AKI and patients without AKI were performed using the Wilcoxon rank-sum test and the chi-square test, as appropriate.

Boxplots were used to illustrate the FO of the different AKI groups (N-AKI, AKI and AKI-RRT) during the first 5 days of ICU stay. The Kruskal-Wallis test was performed to explore whether the three groups differed on individual days. The resulting *p* values were corrected for the multiple test situations with the Bonferroni formula. Post hoc tests, always comparing two groups, were done using the Wilcoxon rank-sum test and *p* values were corrected (Bonferroni).

To visualize the trend of fluid accumulation in reference to the development of AKI, the delta between FO at AKI diagnosis and the FO at each day between 3 days before and up to 3 days after diagnosis of AKI was calculated. Patients were censored at the day of AKI recovery. Means ± standard error (StdErr) of FO were plotted for the whole 7-day period.

A figure with boxplots for the three groups (N-AKI, AKI and AKI-RRT) illustrated the MFO during the ICU stay. Similarly, for the AKI-RRT group, a boxplot described the fluid status at different time points. Survivors and non-survivors were also plotted. The horizontal axis described the median day for the corresponding boxplot event.

To characterize the FO prior to death or ICU discharge, means ± StdErr of all patients who stayed in the ICU for at least 5 days were plotted.

A Kaplan-Meier analysis was performed to predict the time to death for the three AKI groups (N-AKI, AKI and AKI-RRT) separately. The difference between the three groups was tested by a log-rank test. This analysis was restricted to the first 30 days of follow-up; patients who stayed in the ICU for more than 30 days were censored at this time.

An unadjusted logistic regression model was used to illustrate the predicted probabilities of MFO on ICU mortality. For this model, follow-up was restricted to the median time in the ICU (12 days).

Additionally, the previous model was adjusted for AKI status (yes/no during the first 12 days of follow-up) and APACHE II score (at baseline). The predicted probabilities for ICU death were plotted for different APACHE II scores. For both models, the odds ratios (OR) and corresponding 95 % confidence intervals (CI) were reported.

Cox proportional hazard regression analysis was applied to evaluate the time to death. The main predictor was FO_SL_. Hazard ratios (HR) and corresponding 95 % confidence intervals were reported using an unadjusted model as well as a model adjusted for: age, sex, SAPS II, sepsis (yes/no, at admission), mechanical ventilation (yes/no, at admission), diabetes (yes/no, at admission), cardiovascular disease (yes/no, at admission), and hypertension (yes/no, at admission). Only significant variables were shown in the selected models.

*P* values less than 0.05 were considered to be significant. The analysis was conducted with the statistical software SAS, version 9.4 (SAS Institute Inc., Cary, NC, USA).

## Results

A total of 1734 patients from 21 ICUs of nine countries were included in the study. The mean age was 59.2 ± 15.2 years, and 65.3 % were male. The main clinical reasons for admission to ICU were severe cardiovascular (37.6 %), neurologic (12.9 %) and respiratory (11.5 %) problems. A total of 64 % of patients were admitted to the ICU within 24 hours from hospital admission. The mean SAPS II, APACHE II and SOFA scores on admission to the ICU were 39.12 ± 16.99, 17.11 ± 7.66 and 6.63 ± 3.66, respectively. Mean length of stay was 9.5 ± 10.2 days (range 2–11 days) (Table 1).

**Table 1:** Demographics and baseline data

Patient characteristics	All patients (*n* = 1734)	Patients without AKI (*n* = 991) 57.2 %	Patients with AKI^b^ (*n* = 743) 42.8 %	*p* value
Male gender (%)	65.34 %	64.08 %	67.03 %	0.2016
Age in years, mean ± SD	59.23 ± 15.19	57.56 ± 15.48	61.46 ± 14.50	<0.0001
Weight in kg, mean ± SD	75.16 ± 16.89	73.49 ± 15.69	77.38 ± 18.15	<0.0001
- % measured weight	67.99 %	66.20 %	70.39 %	0.0639
Creatinine reference in mg/dl, mean ± SD	1.13 ± 1.02	1.00 ± 0.68	1.29 ± 1.33	<0.0001
- % measured Cr	63.78 %	61.96 %	66.22 %	
Creatinine at baseline in mg/dl	1.14 ± 1.02	1.01 ± 0.78	1.31 ± 1.26	<0.0001
Reason for admission (%)				<0.0001
- Cardiovascular	37.60	36.43	39.17	
- Respiratory	11.53	10.60	12.79	
- Gastrointestinal	9.46	8.27	11.04	
- Trauma	10.32	13.42	6.19	
- Transplant	1.44	0.81	2.29	
- Infections	7.73	5.15	11.17	
- Neurologic	12.92	17.56	6.73	
- Other	8.82	7.77	10.23	
Severity of illness scores (mean ± SD)				
- SAPS II	39.12 ± 16.99	35.04 ± 15.39	44.56 ± 17.50	<0.0001
- APACHE II	17.11 ± 7.66	15.14 ± 6.74	19.74 ± 8.03	<0.0001
- SOFA	6.63 ± 3.66	5.49 ± 3.16	8.15 ± 3.74	<0.0001
Renal pathologies (%)				
- Proteinuria/hematuria	0.98	0.20	2.02	<0.0001
- CKD not on dialysis	8.07	4.94	12.25	
- no CKD	65.22	69.83	59.08	
- renal transplant	1.15	0.40	2.15	
- no data available	24.57	24.62	24.50	
Comorbidities (%)				
- diabetes	21.40	17.05	27.19	<0.0001
- cardiovascular	45.79	43.29	49.13	0.0208
- hypertension	46.94	43.19	51.95	0.0004
Use of NSAIDs (%)	5.59	5.05	6.33	0.2510
Use of ACE-I/ARB (%)	13.09	13.52	12.52	0.5393
Mechanical ventilation (%)				
- on admission	69.61	67.00	73.08	0.0125
- during ICU stay	83.45	79.31	88.96	<.0001
Respiratory support on admission (%)				0.0038
- none	20.88	22.81	18.30	
- non-invasive ventilation	9.63	10.60	8.34	
- invasive ventilation	59.98	56.41	64.74	
Days on mechanical ventilation				
- mean ± SD	6.35 ± 9.18	4.45 ± 6.53	8.87 ± 11.35	<0.0001
- median [IQR]	3.0 [1.0;8.0]	2.0 [1.0; 5.0]	5.0 [2.0; 13.0]	
Sepsis^a^ (%)				
- on admission	23.35	17.25	31.36	<0.0001
- on first AKI day (or admission)	23.24	17.25	31.22	<0.0001
- during ICU stay	34.14	25.43	45.76	<0.0001
Severity of sepsis on admission (%)				
- SIRS	13.01	11.20	15.34	<0.0001
- sepsis	7.11	7.16	7.00	
- severe sepsis	5.72	4.14	7.81	
- septic shock	10.52	5.95	16.55	
RRT in ICU (%)	11.42 %	1.51 %	24.63 %	<0.0001
Death in ICU (%)	12.75 %	5.55 %	22.34 %	<0.0001
Length of ICU stay (days)				
- mean ± SD	9.54 ± 10.19	7.64 ± 7.71	12.07 ± 12.32	<0.0001
- median [IQR]	6.0 [3.0; 12.0]	5.0 [3.0; 9.0]	8.0 [4.0; 15.0]	
Renal status at ICU discharge (%)				
- Dialysis dependent	7.15	1.11	15.21	<0.0001
- Dialysis independent but renal function not returned to baseline	16.96	4.14	34.05	
- renal function returned to baseline	7.67	4.04	12.52	
- normal renal function	68.22	90.72	38.22	

Measured weight: patient’s weight as measured on ICU admission (not estimated). Measured Cr: creatinine from laboratory and not estimated with MDRD

*Abbreviations*: *AKI* acute kidney injury, *SD* standard deviation, *Cr* creatinine, *SAPS II* Simplified Acute Physiology Score II, *APACHE II* Acute Physiology and Chronic Health Evaluation II, *SOFA* Sequential Organ Failure Assessment, *CKD* chronic kidney disease, *NSAID* non-steroidal anti-inflammatory drug, *ACE-I* ACE inhibitor, *ARB* angiotensin receptor blocker, *ICU* intensive care unit, *IQR* interquartile range, *SIRS* Systemic Inflammatory Response Syndrome, *RRT* renal replacement therapy

^a^Sepsis includes: sepsis, severe sepsis, and septic shock

^b^All AKI patients (AKI-RRT included)

**Fig. 1:**
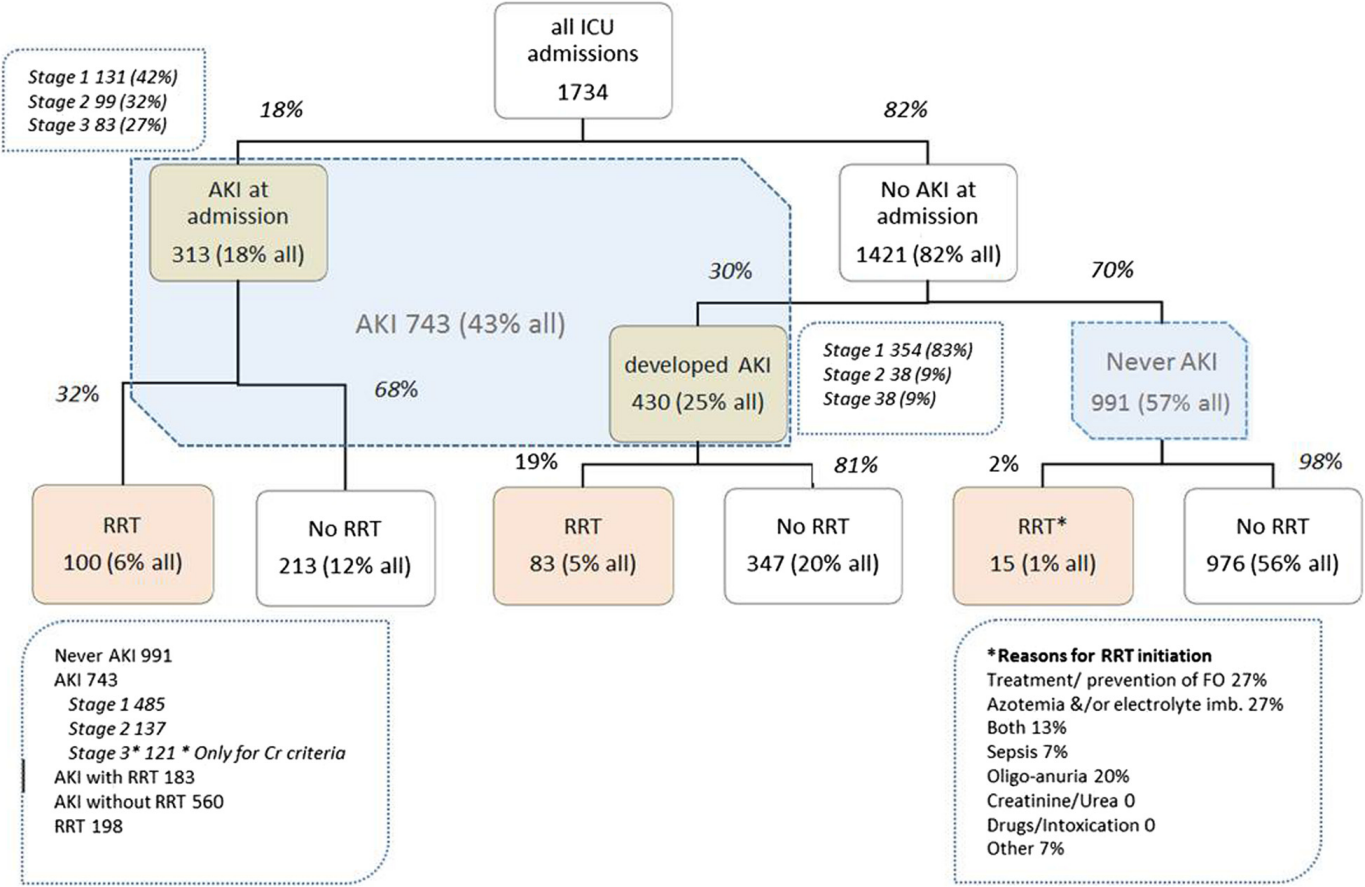
Patient flow chart. *AKI* acute kidney injury, *Cr* creatinine, *RRT* renal replacement therapy

Three hundred and thirteen patients (18 %) had AKI on admission, 430 (25 %) developed AKI during their ICU stay and 183 (25 % of patients with AKI) received RRT (Fig. 1). Among patients with AKI, 66 % had stage I, 18 % stage II and 16 % had stage III.

Patients with AKI (AKI-RRT included) were older, had a higher reference and baseline serum creatinine level, were more often diabetic and septic (both at admission and during the ICU course) and spent more days on mechanical ventilation. Their crude mortality was higher after a longer stay in the ICU (Table 1).

### Fluid accumulation and time course

**Fig. 2:**
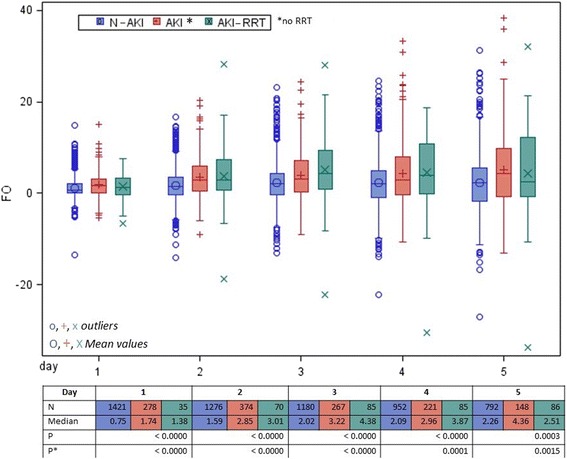
Fluid accumulation for N-AKI, AKI, AKI-RRT patients during the first 5 days following admission. Looking at the first 5 days of ICU stay, the cumulative fluids differed significantly each day among the three groups (N-AKI, AKI and AKI-RRT). (*p* and *p* ^*^ refer to the *p* values of the Kruskal-Wallis test and the correction for the multiple test situation with the Bonferroni test, respectively). There was progressive fluid accumulation in N-AKI and AKI patients. AKI-RRT patients accumulated a similar degree of fluid, followed by a decrease. Patients were daily assigned to the corresponding group (N-AKI, AKI and AKI-RRT). *AKI* patients with acute kidney injury, *AKI-RRT* patients with acute kidney injury treated with renal replacement therapy, *FO* fluid overload, *N-AKI* patients without AKI

There was a progressive fluid accumulation in N-AKI and AKI patients at different time points from admission. AKI-RRT patients had a following decrease (Fig. 2). The three groups showed significantly different degrees of FO at all time points. The differences remained significant after adjusting for the multiple test situation (Bonferroni). (Post hoc test in Additional file 2: Table S1).

**Fig. 3:**
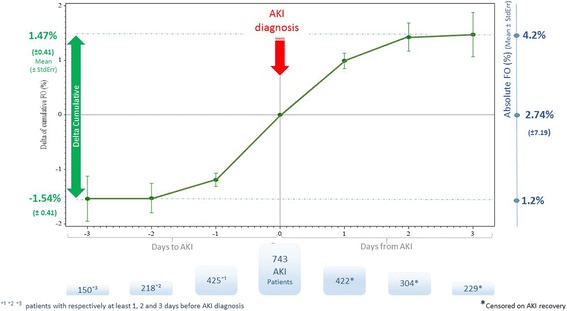
Delta of cumulative fluids pre and post day of AKI. The day of AKI served as a reference point. A backward calculation of cumulative fluid balance was conducted for the 3-day period pre and post day of AKI. Patients were censored on day of AKI recovery. Fluid accumulation up to 3 % occurred within a few days. *AKI* acute kidney injury, *FO* fluid overload, *StdErr* standard error

In AKI patients, fluid accumulation began 3 days prior to AKI diagnosis and continued afterwards, resulting in a total fluid accumulation of 3 % 3 days after development of AKI (Fig. 3).

**Fig. 4:**
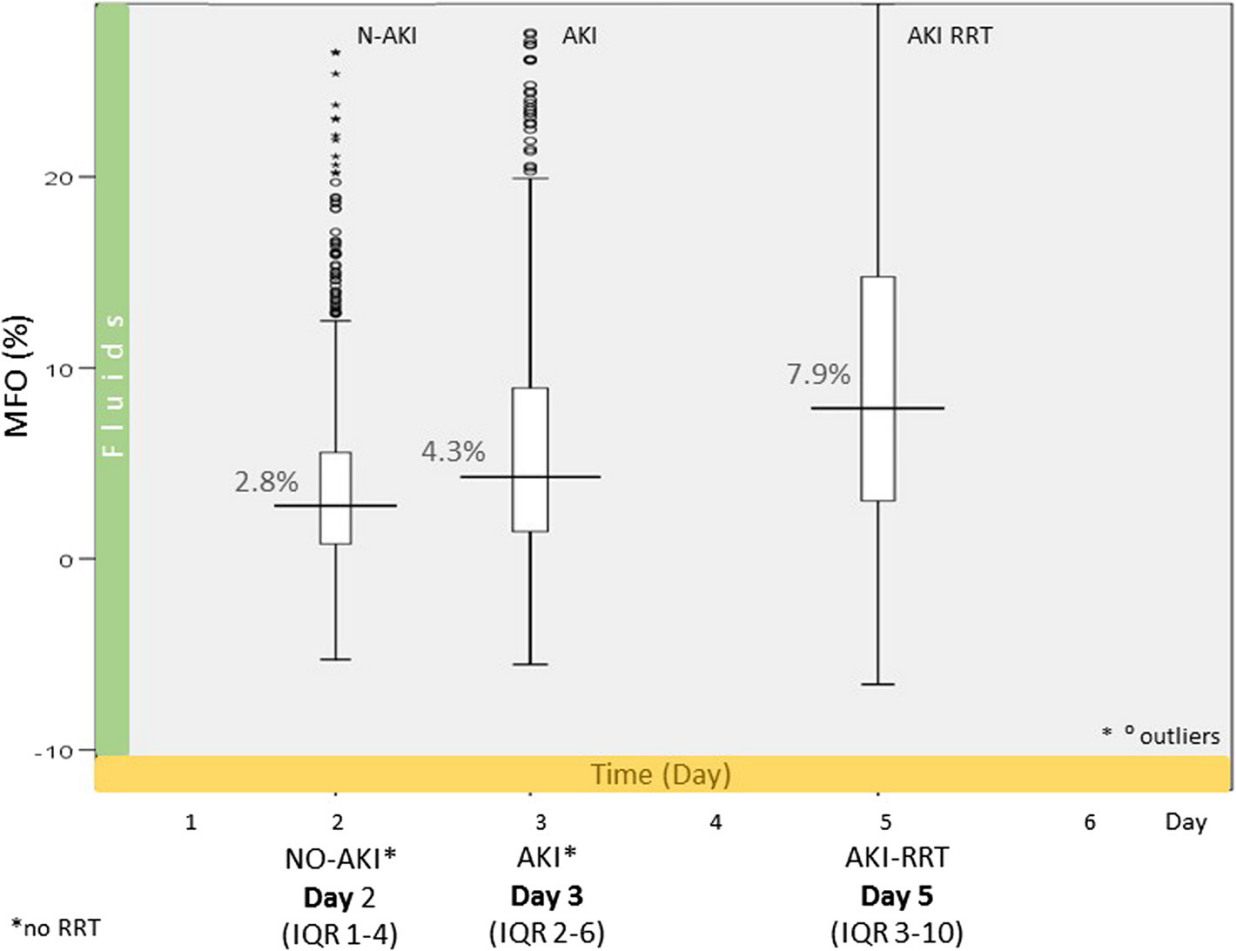
Maximum FO (MFO) during ICU stay in N-AKI, AKI and AKI-RRT patients. MFO was higher in patients with AKI and particularly high in those treated with RRT. The horizontal axis shows the median day when MFO occurred. *AKI* patients with acute kidney injury, *AKI-RRT* patients with acute kidney injury treated with renal replacement therapy, *N-AKI* patients without AKI

**Fig. 5:**
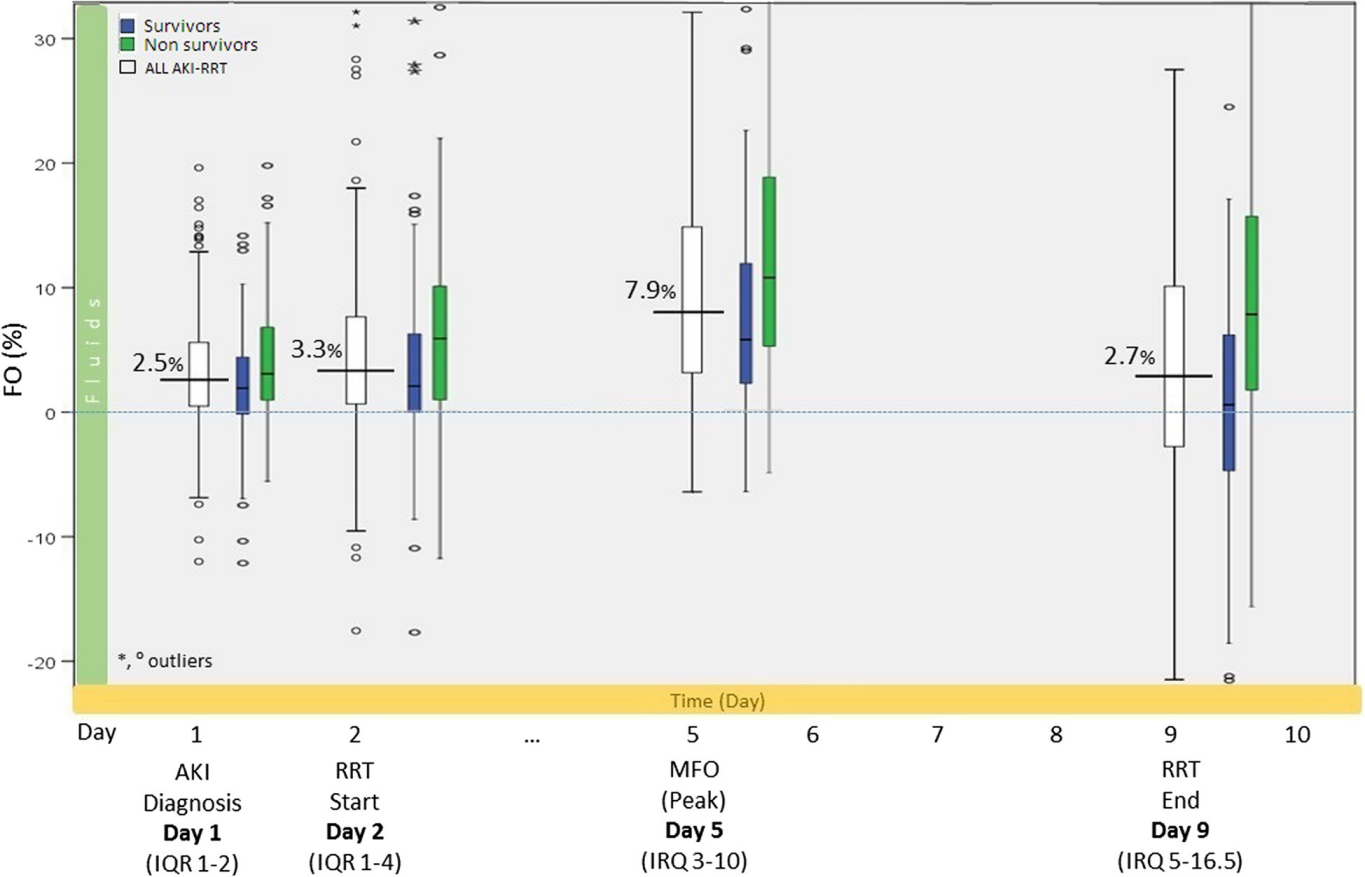
Cumulative fluid balance of AKI patients treated with renal replacement therapy. The cumulative fluid balance of AKI-RRT patients is illustrated at four different time points: (a) day of AKI diagnosis, (b) day of RRT, (c) day of maximum FO (MFO), (d) last RRT day. Cumulative fluid balances were computed also for survivors and non-survivors. The horizontal axis shows the median day when the relevant events occurred. *AKI* acute kidney injury, *AKI-RRT* patients with acute kidney injury treated with renal replacement therapy, *IQR* interquartile range

Maximum fluid overload (MFO) was of 2.8 % of body weight (IQR 0.8–5.6) (on day 2) in N-AKI and 4.3 % (IQR 1.5–9) (on day 3) in AKI (Fig. 4). In AKI-RRT patients, MFO was 7.9 % (IQR 3.1–14.8) (on day 5). In this group, FO was 2.5 % at AKI diagnosis, 3.3 % (IQR 0.6–7.7) at initiation of RRT, and 7.9 % (IQR 3–10) on day 5. On the day when RRT was discontinued (mean day 9), FO was reduced to 2.7 % (IQR 0.6–7.7 %) (Fig. 5).

### Fluid accumulation, mortality and time course

**Fig. 6:**
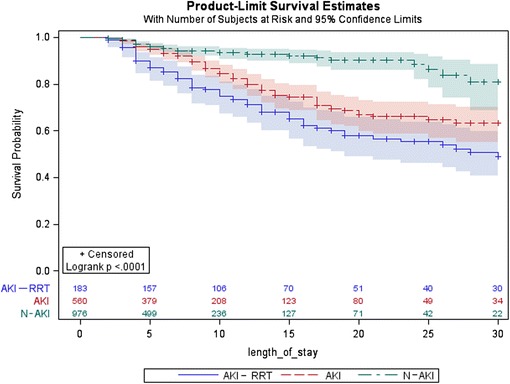
Thirty-day survival of N-AKI, AKI, and AKI-RRT patients. The Kaplan-Meier analysis including the first 30 days of ICU stay indicated a significant survival benefit for patients without AKI (*p* < 0.0001). The AKI-RRT group had the lowest survival rate and AKI patients who did not receive RRT had intermediate survival rates. *AKI* patients with acute kidney injury, *AKI-RRT* patients with acute kidney injury treated with renal replacement therapy, *N-AKI* patients without AKI

The Kaplan-Meier analysis including the first 30 days of ICU stay indicated a significant survival benefit for patients without AKI (*p* < 0.0001). The AKI-RRT group showed the lowest survival rate (Fig. 6). AKI patients had intermediate chances of survival.

**Fig. 7:**
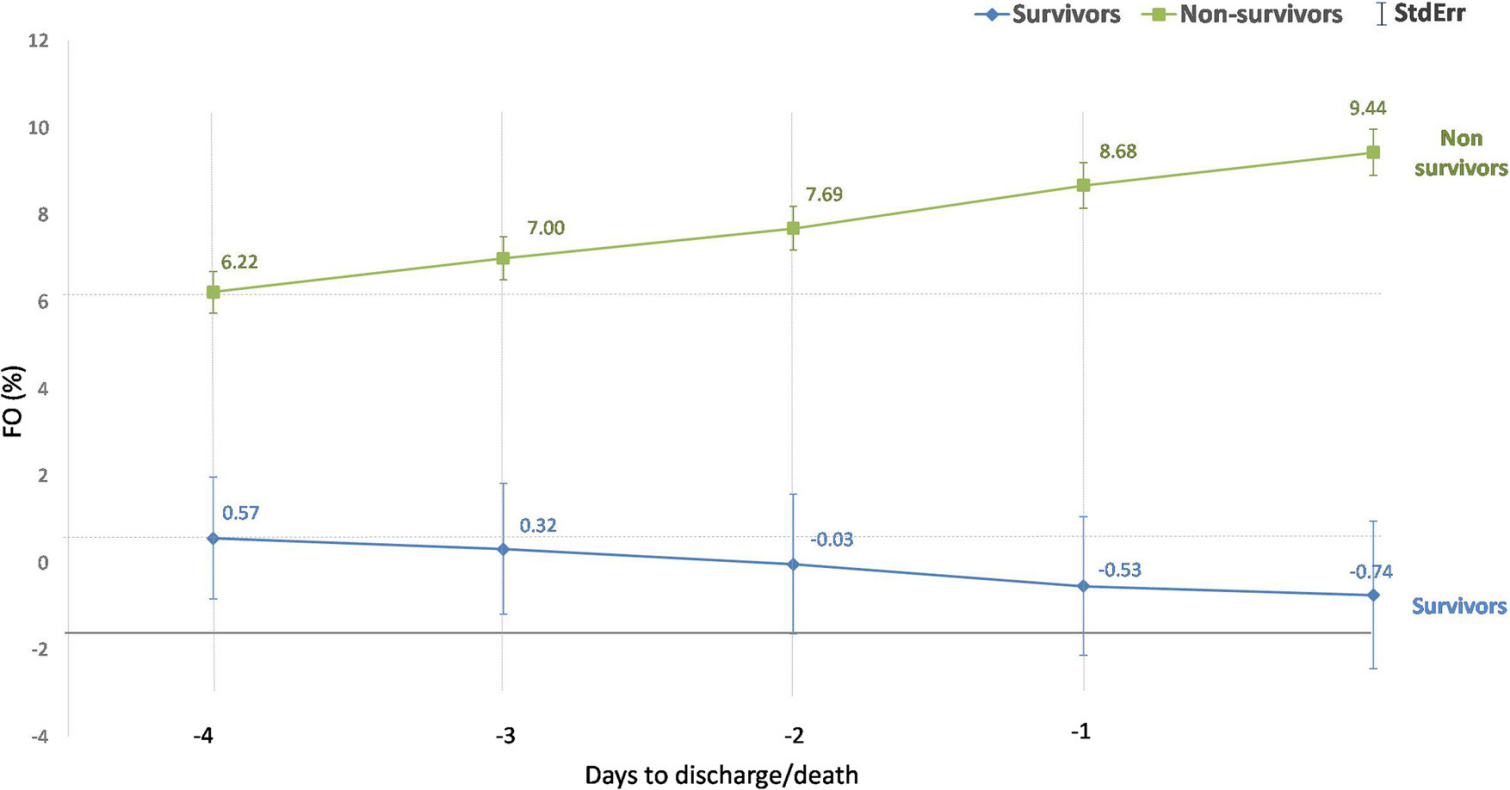
Cumulative fluid balance prior to death or discharge. This analysis includes patients who stayed in the ICU for at least 5 days. Non-survivors (*n* = 156) had progressive fluid accumulation in the 4 days before death whereas cumulative fluid balance decreased in survivors (*n* = 854). *FO* fluid overload, *StdErr* standard error

**Fig. 8:**
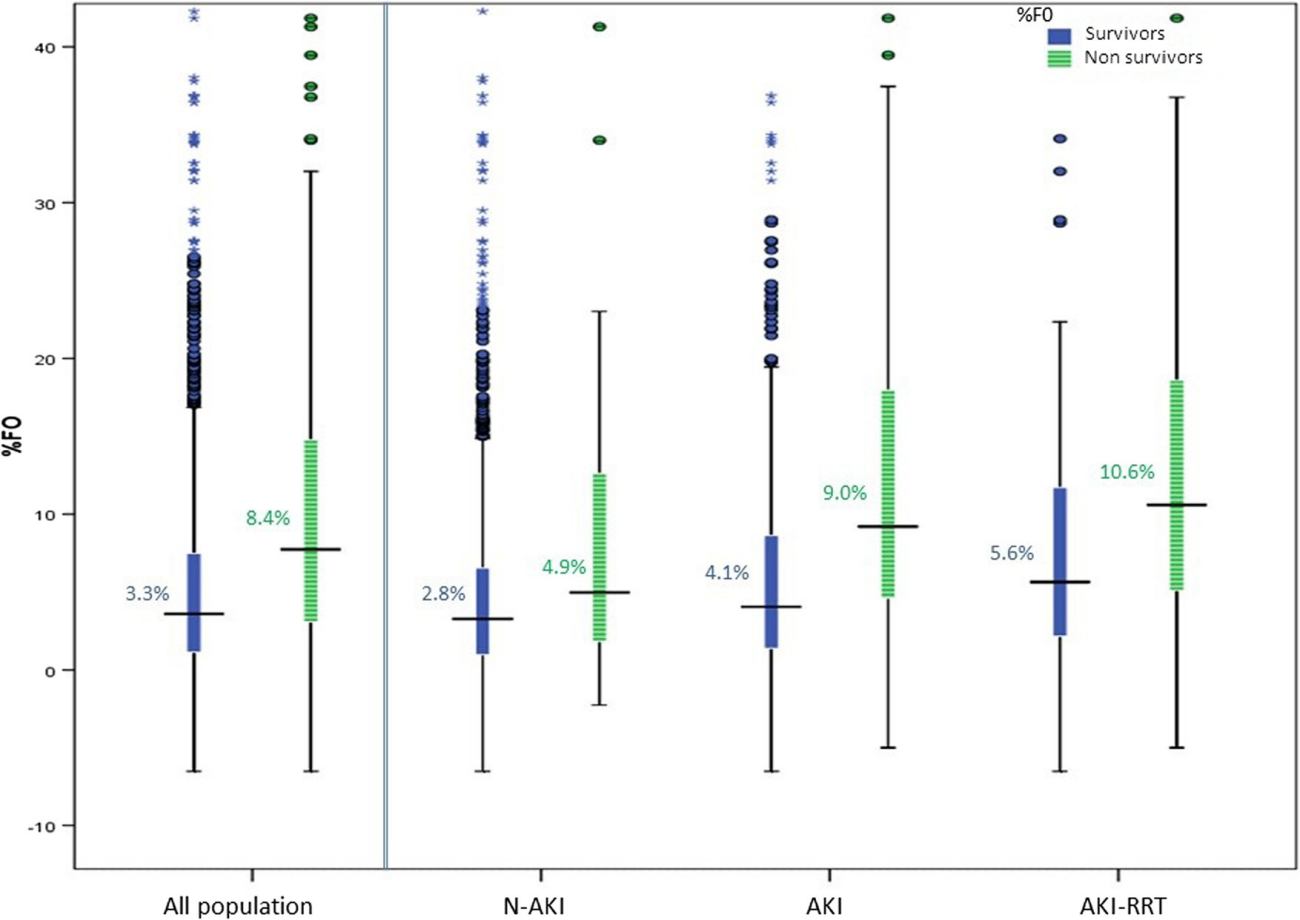
Maximum fluid overload in survivors and non-survivors. Maximum fluid overload (MFO) was calculated for survivors and non-survivors among N-AKI, AKI and AKI-RRT patients. In all cohorts, non-survivors had a higher MFO. *AKI* patients with acute kidney injury, *AKI-RRT* patients with acute kidney injury treated with renal replacement therapy, *N-AKI* patients without AKI

In the total population, non-survivors had significant fluid accumulation in the 4 days prior to death (Fig. 7 and Additional file 2: Table S3). MFO was different between survivors and non-survivors (Fig. 8).

**Fig. 9:**
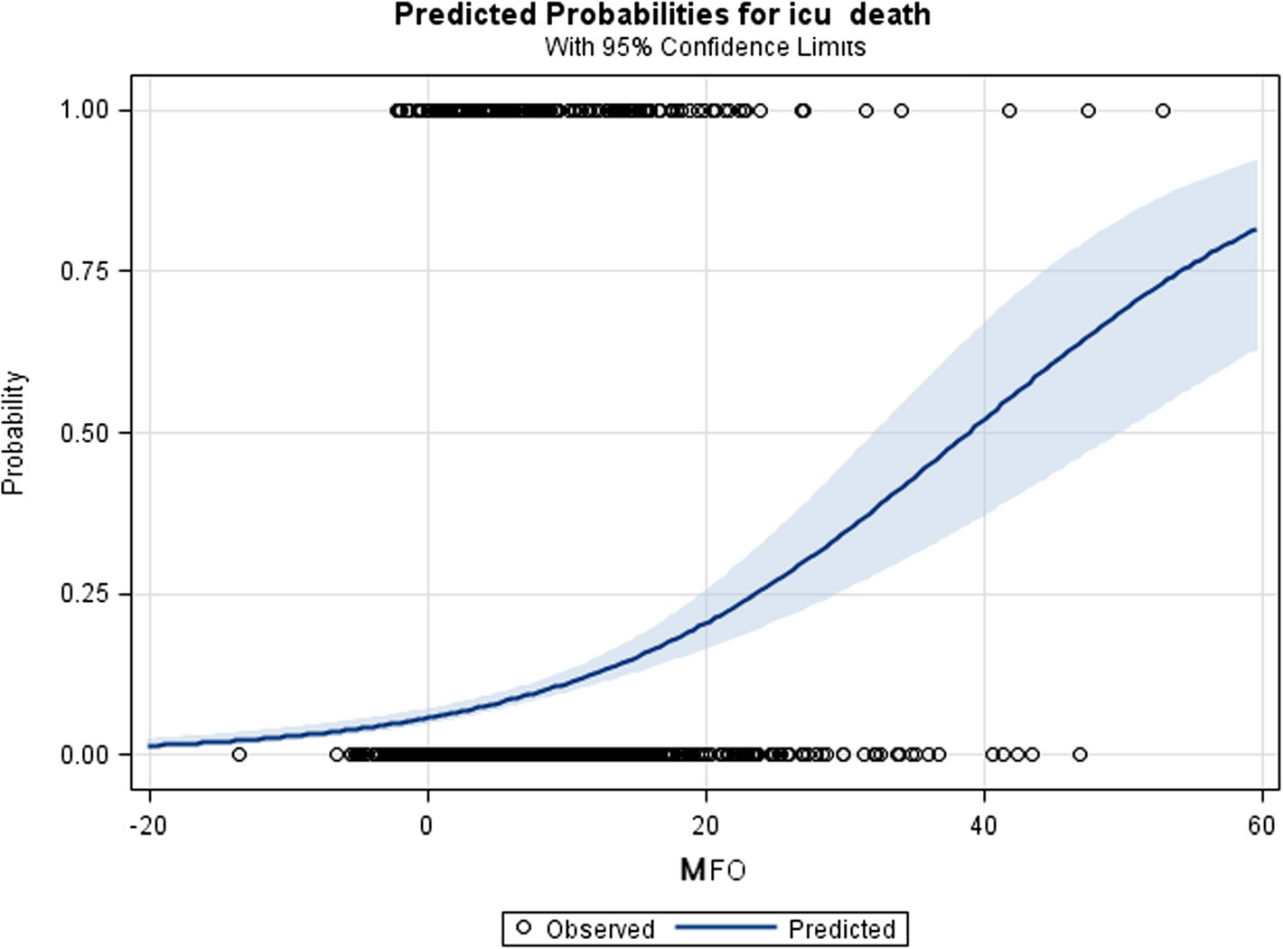
Impact of maximum fluid overload. The risk of death increased exponentially with the magnitude of maximum fluid overload (MFO). In this model, follow-up was limited to the median time in ICU (12 days). *Circles* represent the number of observations of survivors and non-survivors (at the *bottom* and at the *top* respectively)

**Fig. 10:**
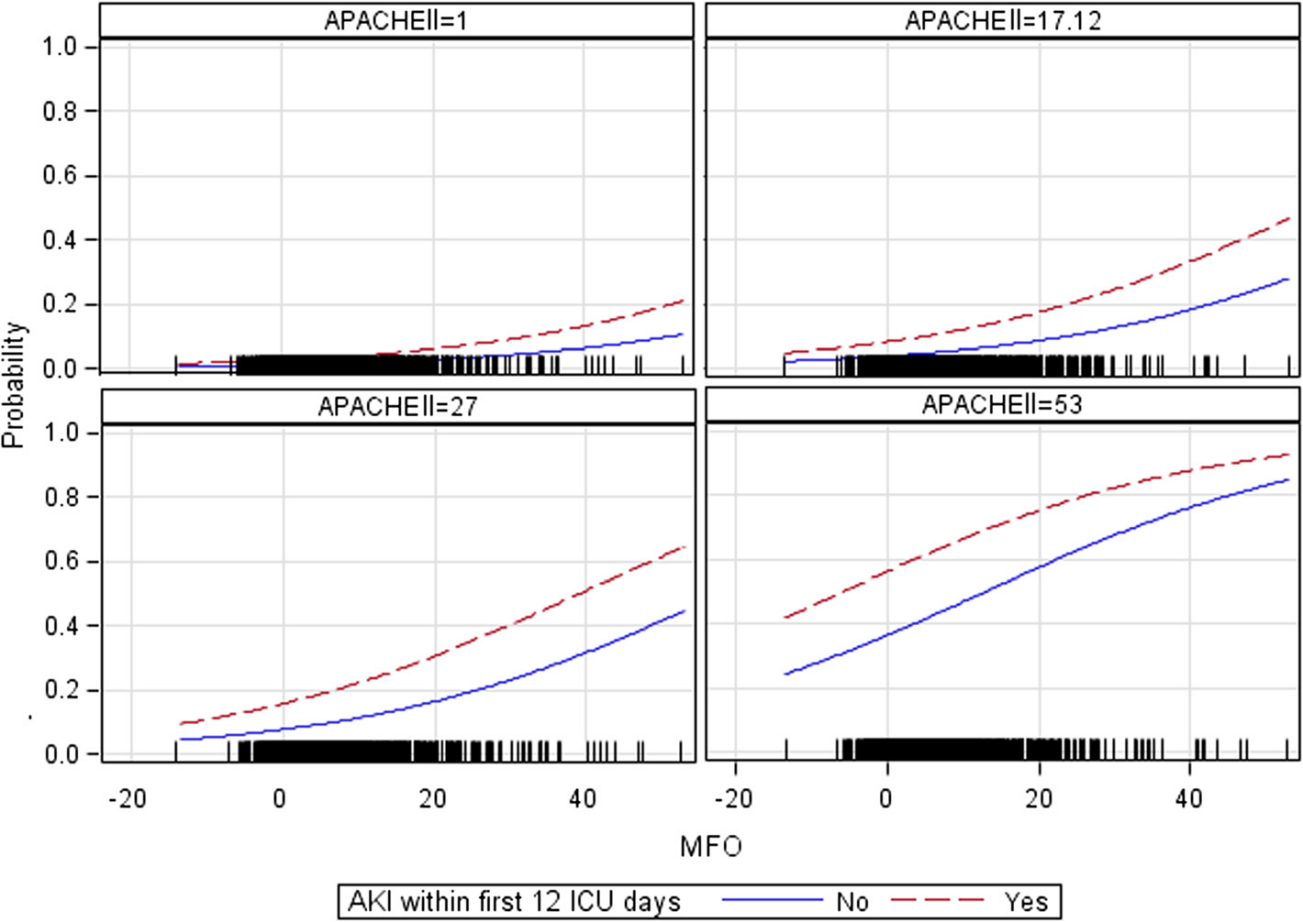
Predicted probability of death adjusted for severity of illness. The figure shows maximum FO (MFO) and predicted probability of death adjusted for APACHE II scores 1, 17.12, 27 and 53. For this model, follow-up was limited to the median time in ICU (12 days). *AKI* acute kidney injury, *APACHE II* Acute Physiology and Chronic Health Evaluation II

A logistic regression showed that MFO was a significant risk factor for mortality. Every 1 % increase of MFO was associated with an OR 1.075 for mortality (CI 1.055–1.095) (Table 2, unadjusted model). There was an exponential relationship between fluid accumulation and predicted probability of death in all patients (Fig. 9). When adjusting the model for APACHE II and AKI status during ICU stay (AKI Y/N), the OR decreased to 1.044 (CI 1.023–1.065) (Table 2, adjusted model). The risk of death related to FO increased with severity of illness as defined by APACHE II score. AKI patients (AKI-RRT included) had a significantly higher risk than patients without AKI (Fig. 10).

**Table 2:** Logistic regression analysis to evaluate the effect of fluid overload on ICU mortality

Unadjusted model		Adjusted model	
	OR (95 % CI)		OR (95 % CI)
MFO	1.075 (1.055–1.095)	MFO	1.044 (1.023–1.065)
		APACHE II	1.077 (1.054–1.101)
		AKI (Yes vs No)	2.247 (1.532–3.295)

*Abbreviations*: *OR* odds ratio, *CI* confidence interval, *MFO* peak fluid overload, *APACHE II* Acute Physiology and Chronic Health Evaluation II, *AKI Yes*: patients met criteria for AKI

### Multivariable analyses

**Fig. 11:**
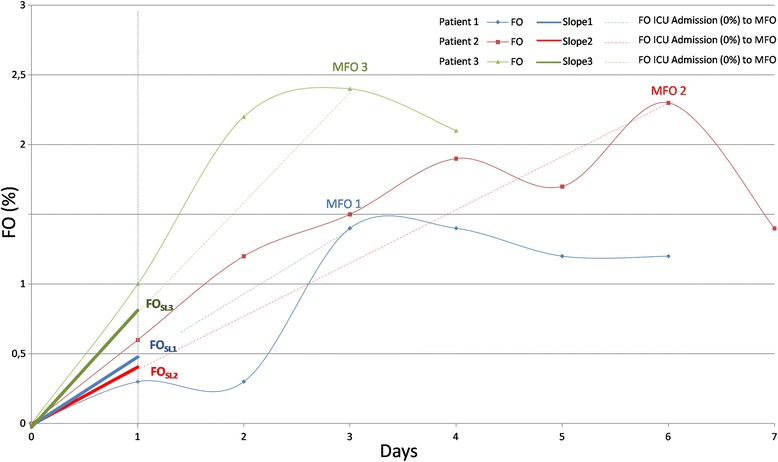
The speed of fluid accumulation. The fluid overload slope (FO_SL_) computed for three generic patients. *Solid line*: FO (%); *dashed line*: ICU admission (FO = 0 %) to maximum FO (MFO) *straight line*; *solid bold line*: FO slope

Cox regression analysis of the velocity of fluid accumulation demonstrated that for every increase of one unit of the FO_SL_, the hazard of death increased significantly by a factor of 1.32 (Table 3). The hazard ratio decreased to 1.28 when adjusting for SAPS II score, sepsis on admission, diabetes, cardiovascular disease and hypertension (Table 3, Fig. 11).

**Table 3:** Multivariable analysis of factors associated with mortality

	Unadjusted model		Adjusted model^a^	
	HR (95 % CI)	*p*	HR (95 % CI)	*p*
FO_SL_	1.32 (1.22–1.43)	<0.001	1.28 (1.18–1.40)	<0.0001
SAPS II			1.02 1.01–1.03	<0.0001
Sepsis			2.01 (1.52–2.65)	<0.0001
Diabetes			1.36 (0.99–1.87)	0.056
Cardiovascular disease			1.43 (1.05–1.94)	0.024
Hypertension			0.67 (0.49–0.92)	0.012

Sepsis was evaluated on admission and included sepsis, severe sepsis and septic shock. Diabetes, cardiovascular disease and hypertension were comorbidities as recorded on admission. Use of diuretics (Y/N) at ICU admission was not statistically significant and intentionally excluded in order to avoid the risk of oversimplification

*Abbreviations*: *HR* hazard ratio, *CI* confidence interval, *FO*_*SL*_ slope of fluid accumulation, *SAPS II* Simplified Acute Physiology Score II

^a^Only significant variables are shown

Similar results were seen after patients were stratified according to N-AKI, AKI, and AKI-RRT status. When adjusting for risk factors, the hazard of death was no longer significant for the N-AKI cohort (*p* = 0.0752) (Table 4).

**Table 4:** Multivariable Cox regression analysis of the impact of fluid accumulation on mortality risk in different subgroups (N-AKI, AKI and AKI-RRT)

	N-AKI		AKI^a^		AKI-RRT	
	HR (95 % CI)	*p*	HR (95 % CI)	*p*	HR (95 % CI)	*p*
Unadjusted model						
FO_SL_	1.28 (1.05–1.55)	0.0131	1.26 (1.11–1.44)	0.0005	1.20 (1.05–1.37)	0.0095
Adjusted (selected) model^b^						
FO_SL_	1.21 (0.98–1.49)	0.0752	1.31 (1.14–1.50)	0.0001	1.21 (1.04–1.42)	0.0137

*Abbreviations*: *N-AKI* patients without AKI, *AKI* patients with acute kidney injury, *AKI-RRT* patients with acute kidney injury treated with renal replacement therapy, *HR* hazard ratio, *CI* confidence interval, *FO*_*SL*_ slope of fluid accumulation

^a^AKI without RRT

^b^Adjusted for the same variables as listed in Table 3

Daily fluid accumulation was a predictor of mortality in the total population and in AKI patients. After adjustment, daily fluid accumulation was not independently associated with a higher risk of death in the N-AKI cohort, even in the context of a higher mean cumulative fluid balance (Additional file 2: Table S2).

## Discussion

Fluid therapy is an integral component of the management of critically ill patients. However, wide variation in clinical practice has been observed, in particular related to type of fluid, rate of administration and methods for assessing fluid responsiveness [16]. Our study shows that ICU patients tended to accumulate fluid in various degrees from the day of admission onwards. In AKI patients, more fluid accumulated between the 3 days prior to the diagnosis of AKI and 3 days later. Despite the fact that all groups accumulated fluid during the ICU stay, AKI-RRT patients showed the highest degree of FO. In this group, FO peaked several days after RRT initiation, which indicates the challenges of removing fluid even with extracorporeal support.

Our results are complementary to the findings of other studies in the literature [17], but also provide new insights into a very complex clinical problem.

Among the overall population as well as the subgroups (N-AKI, AKI and AKI-RRT), non-survivors accumulated more fluid. Moreover, there was a progressive increase in fluid accumulation during the 4 days prior to death. Patients without AKI had a survival benefit (univariate model). The AKI-RRT group had the lowest survival rate during the whole period while AKI patients had an intermediate chance of survival, as previously described in the literature [1, 3, 4, 8].

Furthermore, MFO was an independent risk factor for mortality [OR 1.044 (CI 1.023–1.065)], and the predicted probability of death increased exponentially. AKI patients, including those receiving RRT, were more likely to die than N-AKI. Moreover, the higher the MFO, the wider the difference in mortality between the AKI and N-AKI cohort, which suggests that FO aggravates the patients’ underlying condition [18, 19]. This finding is novel and has not been reported in the literature before. At variance, the PICARD group and others [8, 20–22] concluded that the risk of mortality was proportional to fluid accumulation above a particular cutoff value. Our model predicted the probability of death as a continuum, with an exponential relation to the MFO increase. This suggests that “fluid overload” should be defined as any degree of positive fluid balance rather than a value above an arbitrary cutoff.

We also demonstrated another difference between N-AKI and AKI patients. It seemed that, while AKI independently worsened patients’ outcome, the level of maximum fluid overload was more harmful for patients with AKI than the N-AKI cohort. In addition, severity of illness as defined by APACHE II score was independently associated with mortality. Of interest, the difference in mortality risk between the AKI versus N-AKI cohort tended to increase with higher APACHE II scores.

Our data showed that the velocity of fluid accumulation is also important. Speculating on a possible role of the fluid accumulation velocity, the more rapidly FO occurred, the higher the risk of dying. For any one unit rise of FO_SL_, the probability of death increased 27 times. This finding was significant for all three subgroups in a univariate analysis and remained significant for the AKI cohort but not N-AKI patients after adjustment for other risk factors. This observation may suggest that patients without AKI tolerate a positive fluid balance better during the resuscitation and maintenance phase [23], especially those suffering from sepsis [24, 25].

Based on these results, it appears that, instead of using an absolute fluid accumulation or FO value as a predictor of poor patient outcome, fluid accumulation velocity may have a more physiological rationale and a better statistical power to serve as a tool for fluid assessment.

## Conclusions

In conclusion, this is one of the largest prospective observational studies with data reflecting the real world of clinical practice in the ICU. It is noteworthy that the degree of FO observed in all patients independent of the subgroups was quite low compared to previous studies, which implies that a more careful fluid administration policy has been implemented in most units. Nevertheless, although in most patients FO did not reach the threshold of 5 % and only a small proportion of patients exceeded an FO value of 10 %, risk of mortality was dependent on fluid accumulation in a continuum. Our study also confirms that daily fluid balance tends to be positive in the majority of patients from the day of admission until discharge.

Our study shows that there is an increased risk of dying at any level of FO, and rapidity of fluid accumulation is particularly relevant. Despite these novel findings, it is important to acknowledge some limitations of the study. We recognise the lack of an objective assessment of fluid status at admission and appreciate that the impact of FO and MFO may have been worse in patients who were already fluid overloaded on admission to the ICU. However, independent of fluid status on admission, fluid accumulation occurred inexorably in almost all patients. These findings should trigger further studies, in particular to identify prospective measures to prevent the harmful effects of fluid accumulation, especially if it occurs rapidly.

## Abbreviations

ACE-I, ACE inhibitor; AKI, acute kidney injury; AKI-RRT, patients with AKI treated with RRT; APACHE, Acute Physiology and Chronic Health Evaluation; ARB, angiotensin receptor blocker; BW, body weight; CI, confidence interval; CKD, chronic kidney disease; Cr, creatinine; CRFs, case report forms; CRRT, continuous renal replacement therapy; DoReMi, The Dose Response Multicentre International Collaborative Initiative; ECMO, extracorporeal membrane oxygenation; FO, fluid overload; FO_SL_, slope of fluid accumulation, HR, hazard ratio; ICU, intensive care unit; IQR, interquartile range; MDRD, Modification of Diet in Renal Disease; MFO, maximum fluid overload; MFO/TMFO, velocity of fluid accumulation; N-AKI, patients without AKI; NSAID, non-steroidal anti-inflammatory drug; OR, odds ratios; RRT, renal replacement therapy; SAPS, Simplified Acute Physiology Score; SD, standard deviation; SOFA, Sequential Organ Failure Assessment; StdErr, standard error; TMFO, number of days between ICU admission and day of MFO

## Additional files

Additional file 1: Figure S1. Scores displayed as a pie chart. **Figure S2.** Fluid balance chart. The *red dot* represents the diuretics while the area under the curve is the cumulative fluid. The *green and blue lines* are respectively the total intake and the urine output. (DOCX 250 kb) Additional file 2: Table S1. Post hoc test comparison. **Table S2.** On multivariate Cox regression, the daily cumulative fluid was a predictor of mortality for only the Overall population and AKI patients. After adjusted analysis N-AKI patients were not independently associated with a higher risk of death, even with a higher mean cumulative fluid overload. **Table S3.** FO (%) on ICU discharge for Alive and Death patients. FO (%) for All patients, N-AKI, AKI and AKI-RRT for survivors and non-survivors. (DOCX 19 kb)
