# Supplementary material for: The Dose Response Multicentre Investigation on Fluid Assessment (DoReMIFA) in critically ill patients
Source: Crit Care. 2016 Jun 23;20:196. doi: 10.1186/s13054-016-1355-9 (PMC4918119; doi:10.1186/s13054-016-1355-9)

**Figure S1. Socres calculated from admission data and displayed as pie chart.**


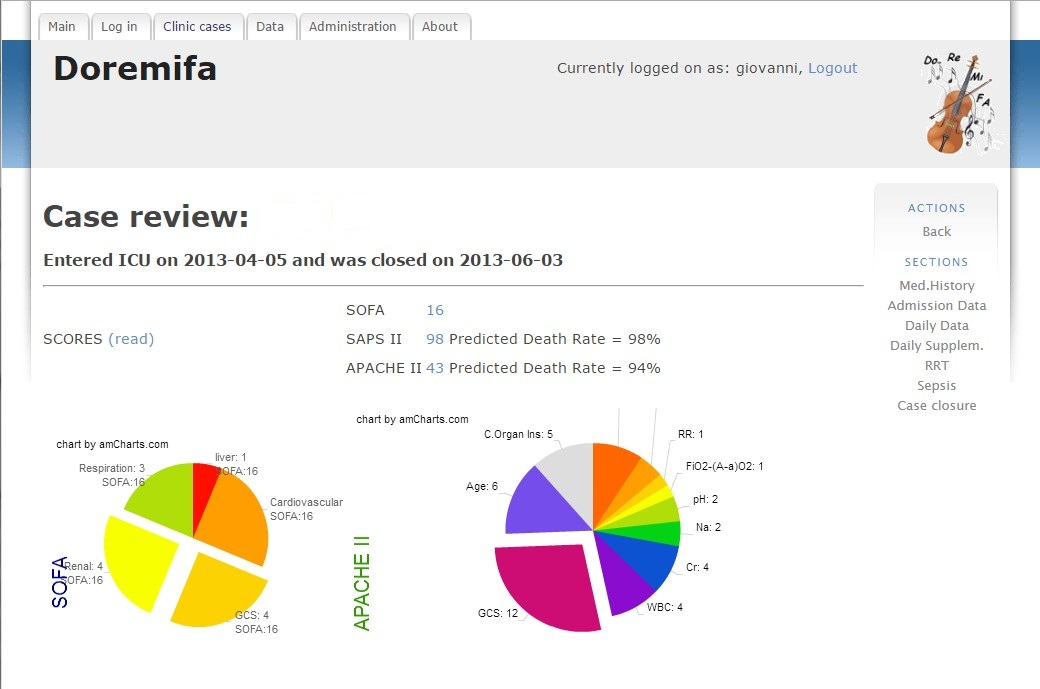


**Figure S2 Fluid balance chart.** The red dot represents the diuretics while the area under the curve is the cumulative fluid. The Green and blue line are respectively the total Intake and the urine output


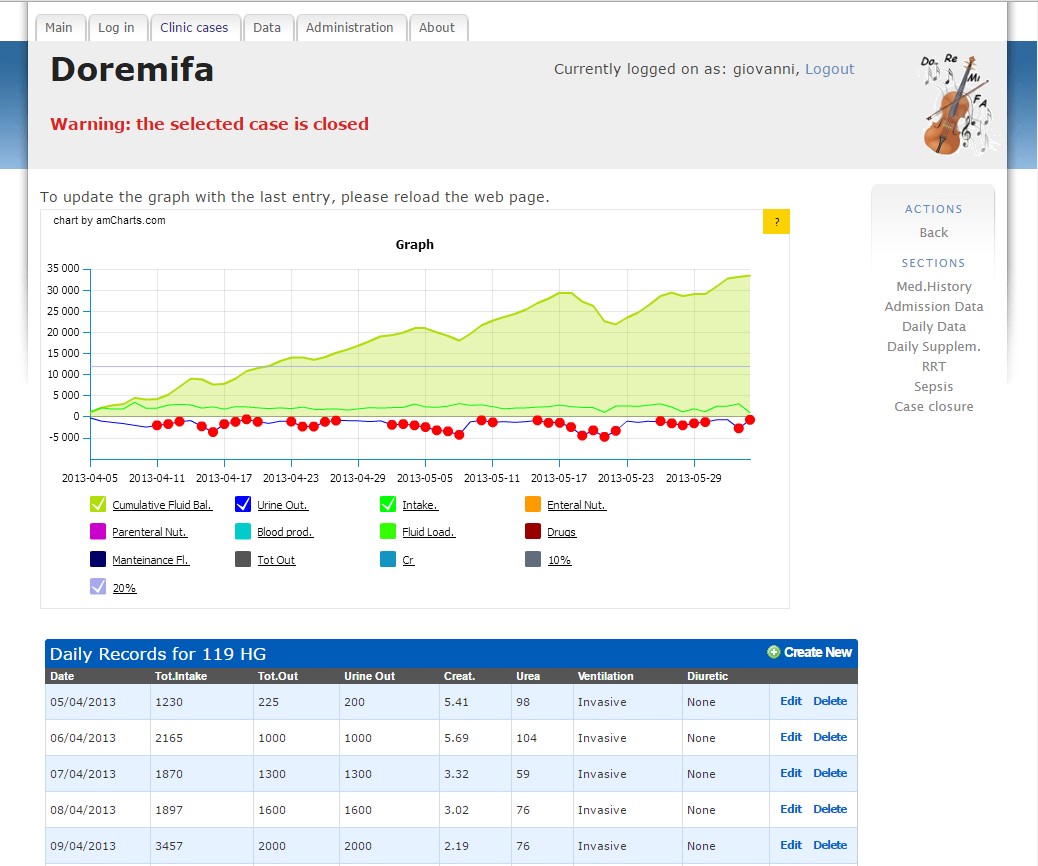

Supplement: Additional file 1: Figure S1. — Scores displayed as a pie chart. Figure S2. Fluid balance chart. The red dot represents the diuretics while the area under the curve is the cumulative fluid. The green and blue lines are respectively the total intake and the urine output. (DOCX 250 kb) [file 13054_2016_1355_MOESM1_ESM.docx]
