# Supplementary material for: The Dose Response Multicentre Investigation on Fluid Assessment (DoReMIFA) in critically ill patients
Source: Crit Care. 2016 Jun 23;20:196. doi: 10.1186/s13054-016-1355-9 (PMC4918119; doi:10.1186/s13054-016-1355-9)
Supplement: Additional file 2: Table S1. — Post hoc test comparison. Table S2. On multivariate Cox regression, the daily cumulative fluid was a predictor of mortality for only the Overall population and AKI patients. After adjusted analysis N-AKI patients were not independently associated with a higher risk of death, even with a higher mean cumulative fluid overload. Table S3. FO (%) on ICU discharge for Alive and Death patients. FO (%) for All patients, N-AKI, AKI and AKI-RRT for survivors and non-survivors. (DOCX 19 kb) [file 13054_2016_1355_MOESM2_ESM.docx]

**Table S1 Post-hoc testComparison of the two groups out of the three groups**

We compared always 2 groups out of the three groups: AKI with RRT, AKI without RRT, and no AKI at the first 5 days of ICU stay. The analysis delivered the following differences on cumulative fluids: AKI without RRT and no AKI patients are significantly different at all 5 days, also after Bonferoni correction. Patients with AKI and RRT compared to patients without AKI are significantly different at day 2,3 and 4. Day 5 is not significant any more after Bonferoni correction.Between AKI-RRT and AKI patients no significant difference could be detected.

1. AKI^1^ versus N-AKI^1^

| **Day** | **1** | | **2** | | **3** | | **4** | | **5** | |
| --- | --- | --- | --- | --- | --- | --- | --- | --- | --- | --- |
| **N** | 1421 | 278 | 1276 | 374 | 1180 | 267 | 952 | 221 | 792 | 148 |
| **Median** | 0.75 | 1.74 | 1.59 | 2.85 | 2.02 | 3.22 | 2.09 | 2.96 | 2.26 | 4.36 |
| **P** | < 0.0001 | | < 0.0001 | | < 0.0001 | | 0.0004 | | 0.0003 | |
| **P*** | < 0.0001 | | < 0.0001 | | < 0.0001 | | 0.0053 | | 0.0045 | |
| **P**** | < 0.0001 | | < 0.0001 | | < 0.0001 | | 0.0070 | | 0.0059 | |

1. AKI-RRT versus N-AKI^1^

| **Day** | **1** | | **2** | | | **3** | | | **4** | | | **5** | |
| --- | --- | --- | --- | --- | --- | --- | --- | --- | --- | --- | --- | --- | --- |
| **N** | 1421 | 35 | | 1276 | 70 | | 1180 | 85 | | 952 | 85 | 792 | 86 |
| **Median** | 0.75 | 1.38 | | 1.59 | 3.01 | | 2.02 | 4.38 | | 2.09 | 3.87 | 2.26 | 2.51 |
| **P** | 0.2953 | | 0.0018 | | | <0.0001 | | | 0.0007 | | | 0.0262 | |
| **P*** | 4.4295 | | 0.0275 | | | 0.0001 | | | 0.0102 | | | 0.3925 | |
| **P**** | 5.9060 | | 0.0367 | | | 0.0002 | | | 0.0136 | | | 0.5234 | |

1. AKI^1^ versus AKI-RRT

| **Day** | **1** | | **2** | | | **3** | | | **4** | | | **5** | |
| --- | --- | --- | --- | --- | --- | --- | --- | --- | --- | --- | --- | --- | --- |
| **N** | 278 | 35 | | 374 | 70 | | 267 | 85 | | 221 | 85 | 148 | 86 |
| **Median** | 1.74 | 1.38 | | 2.85 | 3.01 | | 3.22 | 4.38 | | 2.96 | 3.87 | 4.36 | 2.51 |
| **P** | 0.4712 | | 0.7739 | | | 0.0802 | | | 0.3327 | | | 0.7593 | |
| **P*** | 7.0683 | | 11.6090 | | | 1.2030 | | | 4.9909 | | | 11.3890 | |

^1^ without RRT; P*Bonferoni correct for 15 multiple test; P**Bonferoni correct for 20 multiple test

**Table S2** On multivariate Cox regression, the daily cumulative fluid was a predictor of mortality for only the Overall population and AKI patients. After adjusted analisys NoAKI patients were not independently associated with a higher risk of death, even with a higher mean cumulative fluid overload

| **All patients** | | | **N-AKI** | | | **AKI** | | |
| --- | --- | --- | --- | --- | --- | --- | --- | --- |
| **Parameter** | **HR** | **p** |  | **HR** | **p** |  | **HR** | **p** |
| **Daily cum.** | 1.024 | <.0001 | **Daily cum.** | 1.022 | 0.1388 | **Daily cum.** | 1.022 | <.0001 |
| **APACHE II** | 1.042 | <.0001 | **APACHE II** | 1.097 | <.0001 | **APACHE II** | 1.021 | 0.0270 |
| **Sepsis at admission (Y)** | 1.884 | <.0001 | **Diabetes (Y)** | 1.603 | 0.1297 |  |  |  |
|  |  |  | **Sepsis at admission (Y)** | 4.336 | <.0001 |  |  |  |

Variables considered for the model included: Age, sex, APACHE II, diabetes (Y/N), hypertension disease (Y/N), cardiovascular (Y/N), Mechanical Ventilation admission (Y/N),Sepsis on admission (Y/N). Abbreviations: Daily cum.: daily cumulative balance, APACHEII:Acute Physiology and Chronic Health Evaluation. HR: Hazard Ratio; CI: Confidence Interval.

**Table S3 FO (%) on ICU discharge for Alive and Death patients.** FO (%) for All patients, N-AKI, AKI and AKI-RRT for survivors and non-survivors.

|  | **All patients** | **N-AKI** | **AKI** | **AKI-RRT** |
| --- | --- | --- | --- | --- |
| **Survivors** | 0.92  (-2.93-4.21) | 1.06  (-2.39-3.86) | 0.80  (-3.76-4.79) | 0.34  (-6.12-5.97) |
| **non-survivors** | 6.76  (1.27-14.77) | 2.33  (-1.23-9.13) | 7.62  (2.14-16.07) | 8.81  (2.14-17.82) |

Variables described as Median (IQR)
